# The future of medicine: embracing novelty while sticking to the basics

**DOI:** 10.3325/cmj.2025.66.1

**Published:** 2025-02

**Authors:** Svjetlana Kalanj, Hrvoje Bognar, Barić

**Affiliations:** *Croatian Medical Journal,* Zagreb, Croatia

The preparation of the first *Croatian Medical Journal* (CMJ) issue in 2025 has been a tempting start to the year. We reflected on the previous year by evaluating the scope of the contents we published, and tried to identify the most prominent topics that marked the foregone cycle. A glance at our editorials and cover pages published in 2024 reveals diverse themes. The topics ranged from the application of new technologies in medicine to personalized medicine, investigation of promising biomarkers in neurosurgery, education of health professionals, scientometric pressure in less privileged academic communities, and adaptation of editorial policies in the era of artificial intelligence (AI) (1-6). Not surprisingly, the most impactful publications addressed the application of AI-assisted technologies in science, medicine, health care, and scientific publishing. The meteoric rise of AI with its implications for society has stirred many debates and controversies across all fields, including scientific publishing. AI, although not completely “a new kid on the block,” is revolutionizing the essential societal paradigms and, arguably, catalyzing a civilizational shift. When it comes to medicine, particularly the education of future medical professionals, AI has shown its many positive facets, in particular with regard to the blending of modern AI-based educational methods with the traditional ones (personalized, humanized). This is the topic of a review published in the current issue that examines the usefulness of combining modern and traditional approaches in teaching anatomy – one of the building blocks of medical studies (7). The authors convincingly pinpoint the drawbacks of relying exclusively on the digital form of teaching anatomy and advocate an integrative pedagogic structure that would ensure that students acquire both technical and humanistic skills essential for their work as medical professionals. Today, more than ever, the development of medicine is both challenged and incited by the incorporation of new technologies into everyday practice. If we want to instill intellectual, academic, and empathetic virtues in medical students, we need to be ready for constant adaptations and improvements, bearing in mind the basic concepts of medical discipline.

In addition to screening the topics covered in 2024, we searched the Web of Science to find out who publishes in the *CMJ* and who cites the *CMJ*. As the results show, the *CMJ* is recognized globally, with the majority of papers published during 2024 being authored by scientists with Croatian affiliations (62.5%). The remaining authors hailed from the USA (15%), Bosnia and Herzegovina (11%), Germany and Turkey (both 10%), India (7%), Slovenia (5.5%), Serbia (5.5%), Austria (2.7%), and Italy (1.4%). The publications citing *CMJ* content published during 2024 (mainly research articles, 82%) originated mostly from the USA (27%), followed by Finland, China, Croatia, France, Germany, Italy, and Spain. In general, data extracted for the last year are comparable with previous *CMJ* bibliometric analyses (8). They confirm the continuity of *CMJ* editorial strategies and, more importantly, a highly relevant regional position of our small academic journal.

The *CMJ* persists in pursuing a balance between scientific curiosity and criticism of technological innovations in medicine, not forgetting the very grounds of the medical vocation – compassion and thirst for knowledge that benefits humanity. For this reason, we are thrilled to announce the first *CMJ* issue in 2025 by drawing attention to fundamental questions related to medical education, evaluated from a viewpoint of teaching anatomy (7). This important topic stands as an excellent introduction to the forthcoming round of *CMJ* publications that will present state-of-the-art contributions from the global research community. The *CMJ* editorial team awaits with excitement future advancements in medicine.
